# Highland Barley Polyphenol Delayed the In Vitro Digestibility of Starch and Amylose by Modifying Their Structural Properties

**DOI:** 10.3390/nu14183743

**Published:** 2022-09-10

**Authors:** Xin Ren, Mengyuan Qin, Min Zhang, Yi Zhang, Zhenhua Wang, Shan Liang

**Affiliations:** Beijing Advanced Innovation Center for Food Nutrition and Human Health, National Grain Industry Highland Barley Deep Processing Technology Innovation Center, Beijing Engineering and Technology Research Center of Food Additives, Beijing Technology and Business University, Beijing 100048, China

**Keywords:** highland barley polyphenols, starch, structural characterization, digestibility

## Abstract

Slowing starch digestibility can delay or even prevent the occurrence and development of type 2 diabetes. To explore the hypoglycemic potential of highland barley polyphenols (HBP), this study investigated the structural characteristics and starch digestibility of individual or mixed HBP-starch complexes. The results showed that a V-type structure was formed in HBP-starch complexes through non-covalent bonds, resulting in a decrease in rapidly digestible starch and an increase in resistant starch. Specially, the compounding of HBP extracted by acetone significantly reduced the rapidly digestible starch content in amylose from 41.11% to 36.17% and increased the resistant starch content from 6.15% to 13.27% (*p* < 0.05). Moreover, due to different contents and types of monomer phenols, the HBP extracted with acetone were more effective in inhibiting starch digestion than those extracted with methanol. Ferulic acid and catechin were two key components of HBP. Further results indicated that with the increased content of ferulic acid and catechin (from 1% to 5%), they formed a more ordered structure with amylose, resulting in the lower digestibility of the complex. Collectively, this study suggested that highland barley polyphenols could effectively delay starch digestion by forming a more ordered starch crystal structure. Highland barley polyphenols can be used as functional ingredients in regulating the digestive properties of starchy foods.

## 1. Introduction

Starch is the main component of most cereals. It provides the main glycemic carbohydrates in the human diet [1]. Starch-based foods can be hydrolyzed into glucose by α-amylase, α-glucosidase, or other digestive enzymes and absorbed by small intestinal epithelial cells, increasing postprandial blood glucose [2]. However, the excessive digestion of starch destroys the blood glucose balance, leading to chronic metabolic diseases, especially type 2 diabetes mellitus (T2DM) [3]. In addition, several side effects of clinical drugs aimed at treating T2DM have been reported, including damage to liver and kidney function [4,5]. Therefore, natural foods with slow starch digestion rates are gaining extensive public and research attention in recent years.

Polyphenols are a kind of phytochemicals with benzene rings and multiple hydroxyl chemical structures [6]. Several studies have shown that polyphenols in the food matrix can inhibit starch digestion [7,8]. One reason is that polyphenols inhibit key starch digestive enzymes, including α-amylase and glucosidase [9]. In addition, polyphenols also bind to amylose or amylopectin through hydrogen bonding, hydrophobic interactions, or other molecular forces, subsequently affecting the physicochemical properties and digestibility of starch [8]. On the molecular level, the interaction between various polyphenols and starch can form two types of complexes. One is the noninclusion complex, in which the hydroxyl and carbonyl groups of phenolic compounds interact with starch to form intermolecular aggregates [8], such as maize starch–caffeic acid complexes [10]. The other is a V-type inclusion complex in which phenolic compounds are partially wrapped in the internal hydrophobic helix of starch [11], such as amylose and polymeric proanthocyanidins [7]. Although both amylose and amylopectin could form complexes with polyphenols, the interaction between amylose and polyphenols was better than amylopectin due to its high molecular weight and linear properties [12,13].

Highland barley (*Hordeum vulgare* L.) is the main crop in Tibetan areas of China. It has attracted increased attention in recent years due to its high content of dietary fiber, β-glucan, and polyphenols [12,14,15]. A previous study has demonstrated that highland barley flour had a relatively low starch digestion rate [16]. However, whether highland barley polyphenols (HBP) can interact with starch and further reduce starch digestion has not been reported.

Therefore, in this study, from the mixture HBP-starch complexes to the monomer HBP-amylose complexes, the effects of HBP on starch structure and digestion characteristics were systematically studied. The objectives of this study were (a) to investigate the interaction between HBP and starch; (b) to further explore the effects of HBP on starch digestion properties; and then (c) to reveal the effects of the main monomer phenols in highland barley on the structure and digestion characteristics of amylose.

## 2. Material and Methods

### 2.1. Materials

Highland barley (Beiqing 3) was provided by Shanxi Dongfangliang Life Technology Co., Ltd. (Shanxi, China). Ferulic acid (purity, 99%) and catechin (purity ≥ 98%) were purchased from Shanghai Yuanye Biotechnology Co., Ltd. (Shanghai, China). Pepsin (P700, 250 U/mg), pancreatin (P7545), invertase (I4504, 300 U/mg), and α-glucosidase (10113, 120 U/mg) were purchased from Sigma-Aldrich (St. Louis, Mo, USA). All other chemicals were of analytical grade and purchased from Beijing Banxia Technology Development Co., Ltd. (Beijing, China).

### 2.2. Sample Preparation

#### 2.2.1. Preparation of Highland Barley Polyphenol (HBP)

The HBP was extracted by ultrasound (500 W, 30 min) using an ultrasonic cleaner (KQ5200DB, Beijing Tianlin Hengtai Technology Co., Ltd., Beijing, China) with pure water, 80% methanol, 80% ethanol, and 80% acetone (1:20 *w*/*v*). Highland barley flour was defatted using n-hexane and dried at 40 °C [17]. Then 50 g of defatted highland barley flour was stirred with 250 mL of extracting solution at 200 r/min for 2 h. After being filtered by vacuum suction, the residue was re-stirred for 1 h and repeated twice. The combined extracting solution was concentrated at 40 °C and the remaining aqueous solution was freeze-dried to obtain HBP [12].

#### 2.2.2. Preparation of Polyphenol-Starch Complexes

HBP-amylose complexes, HBP-highland barley starch (HBS) complexes, ferulic acid-amylose complex, and catechin-amylose complex were prepared according to the method described by Amoako and Zheng [12,18] with some modifications. In short, 0.5 g of HBP or monomer phenols were mixed with 5 g of HBS or amylose in 15 mL of 30% ethanol, shaking (180 r/min) for 20 min at 70 °C. After centrifuging at 15,000× *g* for 8 min, the sediments were freeze-dried and sieved through a 100 mesh to obtain polyphenol-starch complexes.

### 2.3. Total Phenolic Content, Total Flavonoid Content and Total Antioxidant Activity

The total phenolic content and total flavonoid content were determined according to previously reported methods [19,20]. The total phenolic content was expressed as µmol of gallic acid equivalents/g of samples. The total flavonoid content was expressed as µmol of catechin equivalent/g of samples.

The ferric reducing antioxidant power (FRAP) and Trolox equivalent antioxidant capacity (TEAC) was measured using the total antioxidant capacity kit (A015-3-1 and A015-2-1, Nanjing Jiancheng Bioengineering Research Institute, Nanjing, China) according to the manufacturer’s instructions. The FRAP results were described as µmol Fe^2+^ equivalents/g of samples, and the TEAC results were described as µmol Trolox equivalents/g of samples.

### 2.4. Binding Ability

Following Zheng [18], the binding ability of the polyphenol–starch complexes was determined by measuring the content of polyphenols in supernatant before and after binding. The solution before the binding was mimicked by dissolving 0.5 g of polyphenol in 15 mL of ethanol and shaking (180 r/min) for 20 min at 70 °C. The binding ability was calculated with Equation (1):

(1) $$\mathrm{Binding}\;\mathrm{ability}=\left(1-\frac{\mathrm{polyphenol}\;\mathrm{content}\;\mathrm{after}\;\mathrm{binding}}{\mathrm{polyphenol}\;\mathrm{content}\;\mathrm{before}\;\mathrm{binding}}\right)\times 100\%$$

### 2.5. Scanning Electron Microscope (SEM)

The microstructure of the samples was analyzed using an SEM (SU8020, Hitachi, Japan) according to previous methods [21,22]. Briefly, the samples were spread on a strip of double-sided carbon-coated tape, sprayed with a thin layer of gold, and finally placed in the SEM chamber. The microstructures of the samples were captured at 3000× magnification with an SEM operating at accelerating voltage of 5 kV or 3 kV.

### 2.6. Fourier Transforms Infrared (FT-IR) Spectroscopy

The chemical bonds of the samples were determined with an FT-IR spectrometer (Nicolet IS10, Madison, WI, USA). The samples were equilibrated at room temperature for 24 h, then mixed with KBr and pressed into thin slices before measurement. Scanning wave numbers ranged from 400 to 4000 cm^−1^ by accumulating 32 scans per spectra at 4 cm^−1^ [23]. 

### 2.7. X-ray Diffraction (XRD)

The crystal types of the samples were characterized with an X-ray diffractometer (D8 ADVANCE, BRUCKER, Salbrucken, Germany) following Zheng et al. [18]. The samples were scanned over the range of 4°–40° 2θ angles with a step width of 0.026° (2θ), and a speed of 5°/min.

### 2.8. Differential Scanning Calorimeter (DSC)

The thermal properties of samples were measured using a DSC (DSC-60 Plus, Shimadzu, Columbia, CT, USA) based on He’s method [1] with some modification. In short, 3 mg of samples and 9 mg of deionized water were placed into an aluminum crucible, equilibrating at room temperature for 24 h. The pan was then scanned from 30 °C to 130 °C at a heating rate of 10 °C/min. The data were analyzed with DSC software from TA instruments (New Castle, PA, USA).

### 2.9. In Vitro Gastrointestinal Digestion 

The digestibility of samples was analyzed according to the method described by Englyst et al. [24]. Briefly, 0.3 g of sample and 10 mL of pepsin solution (0.5%, *w*/*v*) were placed in a 50 mL test tube, incubating in a shaking water bath at 37 °C for 30 min. After the pH was adjusted to 5.5 with 0.1 mmol/L acetate buffer solution, the active mixed enzyme solution containing 0.75 mL amyloglucosidase (1200 U/mL) and 1 mL invertase (3000 U/mL) was added, shaking at 37 °C. At specified time points (0, 20, 120 min) during digestion, 0.2 mL of samples were taken out and mixed with 4 mL of anhydrous ethanol to deactivate the enzymes. After centrifugation at 1500× *g* for 5 min, the glucose contents in the supernatant were measured by a glucose assay kit (A154-1-1, Nanjing Jiancheng Bioengineering Research Institute, Nanjing, China).

The rapidly digestible starch (RDS), slowly digestible starch (SDS), and resistant starch (RS) contents were calculated using the following formula:

(2) $$\mathrm{RDS}=\left(G_{20}-G_{0}\right)\times 0.9$$

(3) $$\mathrm{SDS}=\left(G_{120}-G_{20}\right)\times 0.9$$

(4) RS=TS−RDS−SDS

where G_0_, G_20_, and G_120_ represent the content of glucose released at 0, 20, and 120 min, respectively. TS represent total starch. And 0.9 means the conversion factor of glucose from starch.

### 2.10. Statistical Analysis

All data were expressed as mean ± standard deviation (mean ± SD) for at least three replicates per sample. ANOVA and Tukey’s test were performed using SPSS 17.0 (IBM, Chicago, IL, USA). All graphs were produced using Origin 2018 (Origin Lab, Northampton, CA, USA). The statistical significance was set at *p* < 0.05.

## 3. Results and Discussion

### 3.1. Phenolic Compounds and Antioxidant Activity

The phenolic compounds and antioxidant activities in highland barley flour extract by different solvents are shown in Table 1. The phenolic compound contents and antioxidant activity were highest in the 80% acetone extraction solution, followed by those in 80% methanol and 80% ethanol extraction, and the lowest in pure water extraction. There were no significant differences in phenolic compound content or antioxidant activity between 80% methanol and 80% ethanol. A similar result was also reported in the extraction of phenolic compounds from litchi pulp [25]. However, Zhao et al. [26] found that the 80% methanol extract showed higher O_2_^·−^, DPPH^·^, and ABTS^·+^ scavenging activities than the 80% ethanol. Therefore, the methanol and acetone solvents were used to extract HBP in subsequent experiments in this study.

### 3.2. Binding Ability of HBP-Starch Complexes

The binding abilities of HBP and starch are displayed in Table 2. The binding abilities of 80% acetone extracted HBP (AHBP)-amylose complexes and AHBP-HBS complexes were significantly higher than that of the 80% methanol extracted HBP (MHBP)-HBS and MHBP-amylose complexes. This finding indicated that different extraction methods of polyphenols could affect the binding capacity of the complexes. The binding ability of AHBP to form complexes was significantly higher than that of MHBP. In addition, the binding ability of the AHBP-amylose complexes was higher than that of the AHBP-HBS complexes. This could be attributed to the fact that amylose was easier to form complexes with polyphenols than amylopectin [27]. The amylose content in HBS varies from 0–40% due to different varieties [23].

### 3.3. Complexation between HBP and Starch

The formation of polyphenol–starch inclusion complexes can be demonstrated by various techniques including XRD, FT-IR, and so on [8,28]. On the FT-IR spectrum, the different positions of absorption peak corresponding to different chemical bonds [29]. The changes in the corresponding chemical bonds can be explored through the changes in the positions of the characteristic absorption peaks. In the present study, we investigated the differences in the spectra of various samples before and after the combination of starch and polyphenols to explore the effect of polyphenols on starch structure.

**Figure 1:**
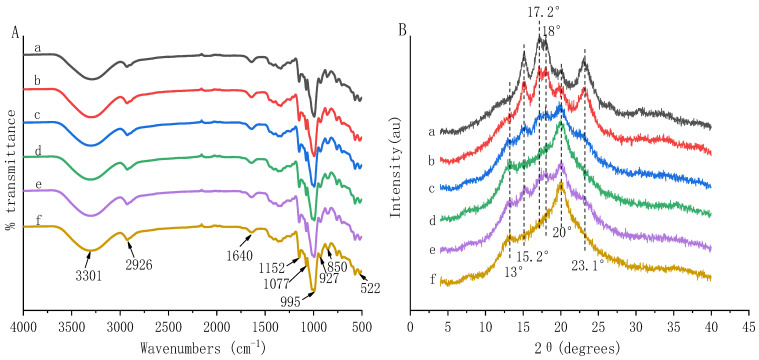
Infrared spectra (**A**) and X-ray diffraction pattern (**B**) of highland barley polyphenol-starch complexes extracted with different solvents. (a) Amylose; (b) Highland barley starch (HBS); (c) 80% methanol extracted highland barley polyphenols (MHBP)-amylose complexes; (d) 80% acetone extracted highland barley polyphenols (AHBP)-amylose complexes; (e) 80% methanol extracted highland barley polyphenols -highland barley starch (MHBP-HBS) complexes; (f) 80% acetone extracted highland barley polyphenols-amylose complexes (AHBP-HBS) complexes.

The FT-IR spectra of HBP-starch complexes are shown in Figure 1A. All samples showed typical bands and peaks in the FT-IR spectra. They include the band around 3100 to 3500 cm^−1^, which belonged to the stretching vibration of -OH and the absorption of hydrogen bonds; the spectral peak near 2926 cm^−1^, which was assigned to the anti-symmetrical stretching vibration of −CH_2_ [30]; and the absorption peak around 1640 cm^−1^, which was attributed to the amorphous region vibration [1]. There were still many absorption peaks at 1800~600 cm^−1^, mainly attributed to the vibration of ether bonds, ester bonds, and ether bonds [30]. Compared with the native starch, all the HBP-starch complexes showed the same characteristic peaks, indicating no new covalent bond formation between HBP and amylose or HBS. This result suggested that HBP was more likely to interact with starch through non-covalent interactions, such as hydrogen bonds and hydrophobic interaction [12]. A similar result has been reported that grape seed proanthocyanidins and potato starch might interact through noncovalent bonds, especially hydrogen bonds [30].

The bands of 1047 and 1022 cm^−1^ in the FT-IR spectrum are related to the crystalline and amorphous regions. The ratio of 1047/1022 cm^−1^ peak intensities can represent the ordered structure of starch crystals [31]. Generally, a higher ratio indicates a higher degree of order [31,32]. The results of 1047/1022 cm^−1^ were calculated and are shown in Table 2. In this study, the ratios of 1047/1022 cm^−1^ of HBP-starch complexes were higher than those of amylose or HBS, indicating that a more ordered crystal structure was formed in HBP-starch complexes.

XRD is an analytical technique for studying the molecular structure of crystals and has been recognized as an effective method for studying the interactions between small-molecule ligands and macromolecular receptors at the molecular level, such as polyphenol–starch complexes [33,34]. The X-ray diffraction patterns of HBS, amylose, and HBP-starch complexes were measured (Figure 1B) in this study. Amylose can form a left-handed single-helix structure, allowing small guest molecules such as polyphenols to enter the helical cavity through hydrophobic interactions, forming an inclusion compound and presenting standard V-type diffraction patterns with peaks at 2θ = 13° and 20° [11,35]. Thus, the presence of polyphenol-starch complexes can usually be demonstrated by unique XRD patterns.

In the present study, amylose and HBS exhibited strong characteristic peaks at 15.2° and 23.1° and unresolved doublet peaks at 17.2° and 18°, showing a typical A-type crystal structure [36]. Compared with amylose and HBS, X-ray diffraction patterns showed that new peaks appeared and old peaks disappeared after compounding with HBP. Specifically, 2 new diffraction peaks at 13° and 20° were observed in HBP-starch complexes, which were similar to previous research results [35,37]. They were characteristic peaks of V-type complexes, suggesting the formation of V-type complexes between HBP and starch [11]. The V-type complex is a highly ordered and stacked molecular structure [38]. This result was consistent with the FT-IR analysis finding that a more ordered crystal structure formed in the HBP-starch complexes (Table 2). Moreover, the diffraction peaks at 13° and 20° of the AHBP-starch complexes were higher than those of MHBP-starch complexes. And the diffraction peaks at 15.2° and 17.2° were also observed in MHBP-amylose and MHBP-HBS complexes, indicating the formation of A-type and V-type complexes simultaneously between MHBP and starch [11,36]. These might be related to the specific monomer phenol composition and content of polyphenols extracted with different solvents.

### 3.4. Microstructure of HBP-Starch Complexes

**Figure 2:**
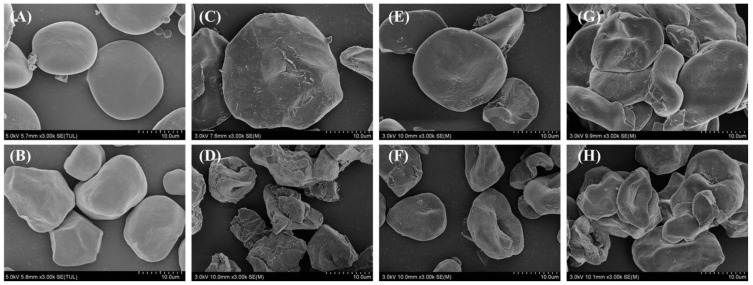
Scanning electron micrographs of starch and polyphenols-starch complexes. (**A**) Highland barley starch (HBS); (**B**) Amylose; (**C**) Gelatinized highland barley starch (HBS); (**D**) Gelatinized amylose; (**E**) 80% methanol extracted highland barley polyphenols-highland barley starch (MHBP-HBS) complexes; (**F**) 80% methanol extracted highland barley polyphenols (MHBP)-amylose complexes; (**G**) 80% acetone extracted highland barley polyphenols-amylose complexes (AHBP-HBS) complexes; (**H**) 80% acetone extracted highland barley polyphenols (AHBP)-amylose complexes.

The SEM images of starch and HBP-starch complexes are shown in Figure 2. The non-gelatinized HBS (Figure 2A) appeared to be smooth, flat, and oval, with uniform size and a small proportion of small particles. The non-gelatinized amylose (Figure 2B) was a multi-angle ellipsoid with a smooth particle surface. The original shape of the amylose disappeared after gelatinization, showing a distinct fragmentation (Figure 2D). Meanwhile, the gelatinized HBS still presented an oval structure, but the surface of some granules was damaged (Figure 2C). After the addition of HBP (Figure 2E–H), the damage to the starch granule structure was alleviated. It could be observed that the starch had apparent agglomeration with a relatively smooth surface. This result demonstrated that the complexation of HBP with starch could form a more compact and stable starch granule structure that was more resistant to gelatinization. Similar results were observed by Li et al. [39]. In addition, compared with the MHBP-starch complexes, a more pronounced aggregation was observed in AHBP-starch complexes, which indicated that AHBP-starch complexes might be more effective in inhibiting starch gelatinization than MHBP-starch complexes. 

### 3.5. In Vitro Digestibility of HBP-Starch Complexes

The starch digestibility of HBP-starch complexes extracted with different solvents is shown in Table 2. RDS could rapidly increase blood glucose levels and insulin secretion, leading to diabetes-related symptoms. RS can escape enzymatic hydrolysis in the small intestine without causing elevated blood glucose [40,41]. Hence, it is crucial to increase the content of RS and reduce RDS in starchy foods to maintain the metabolic balance of blood glucose. In the present study, compared with corresponding amylose and HBS, the content of RDS decreased and RS increased in HBP-amylose and HBP-HBS complexes, indicating slow starch digestion. This favorable change could be attributed to the interaction of HBP with amylose or HBS, affecting the structure of starch and forming a V-type structure [1]. The V-type inclusion complex had a more compact structure, which could reduce the active site of α-amylase [38]. Moreover, its high order could increase the resistance to α-amylase, thus inhibiting starch digestion [11]. It has also been reported that one of the reasons for the higher content of RS in polyphenol-starch complexes was the release behaviors of polyphenol from complexes during gastrointestinal digestion [18]. However, the release of HBP during in vitro digestion in this study needs further exploration. 

Notably, the compounding of AHBP had a significant effect on the decrease of RDS content and the increase of RS content in amylose but had little effect on HBS. This might be attributed to the interaction between polyphenols and starch was more likely to be enhanced through extensive hydrogen bonding and hydrophobic interactions in starch with high amylose content [12]. Besides, the effect of AHBP-amylose complexes on starch digestion was greater than that of MHBP-amylose complexes. It was proved again that the composition and content of specific monomer phenols could significantly affect the properties of polyphenol-starch complexes [42,43]. 

### 3.6. Characterization of Amylose Compound with Main Monomer Phenol

It has been reported that different types and contents of polyphenols have different inhibitory effects on starch digestion [42,43]. In the present study, there were also some notable points, including (a) the binding ability and peak intensity at 13° and 21° of AHBP-starch complexes were higher than those of MHBP-starch complexes (Table 2 and Figure 1B); (b) more pronounced aggregation was observed in AHBP-starch complexes compared with the MHBP-starch complexes (Figure 2); (c) the RDS content was significantly decreased and RS content was significantly increased in AHBP-amylose complexes, but there was no significant difference in MHBP-amylose complexes (Table 2). These results might be attributed to the difference in the content and type of monomer phenol extracted by 80% acetone and 80% methanol. 

Thus, we further analyzed the main composition of HBP. The results showed that the main monomer phenols in highland barley were ferulic acid, catechin and p-coumaric acid (Table 1). The contents of ferulic acid and catechin extracted with 80% acetone were significantly higher than those extracted with 80% methanol. But there was no significant difference in p-coumaric acid. Therefore, we then explored the effects of different contents of ferulic acid and catechin on the structure and digestion characteristics of amylose by XRD, DSC and in vitro simulated gastrointestinal digestion.

#### 3.6.1. In Vitro Digestibility of Amylose Compound with Main Monomer Phenol

**Figure 3:**
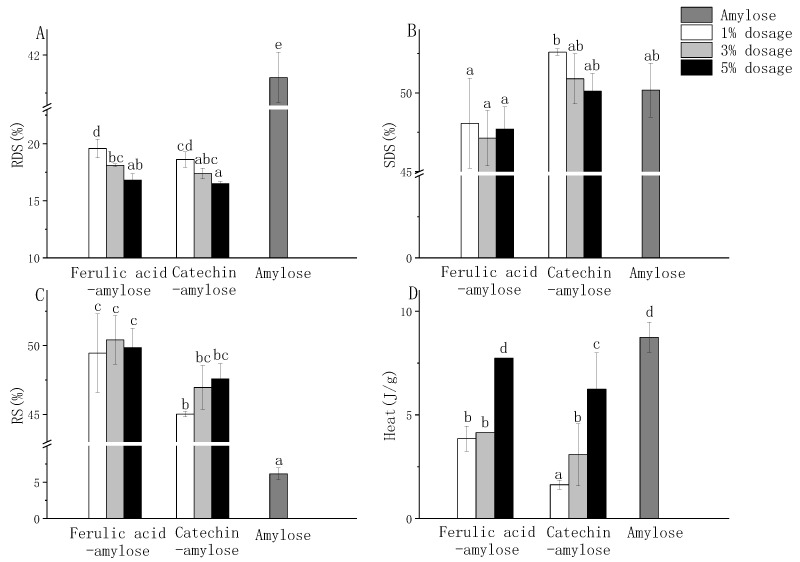
Effect of different standard and addition amounts on starch digestibility (**A**–**C**) and thermal properties (**D**) of polyphenol-amylose complex. Different letters represent significantly different (*p* < 0.05).

The effects of different types and additions of main monomer phenol on amylose digestibility are shown in Figure 3A–C. The RDS content had a clear dose-dependent relationship with the contents of ferulic acid and catechin. The RDS content in 5% ferulic acid-amylose and 5% catechin-amylose complex was significantly lower than that in their corresponding 1% added complex (Figure 3A). In addition, there were significant differences in the SDS and RS contents between 1% ferulic acid-amylose complex and 1% catechin-amylose complex (Figure 3B,C). This validated our hypothesis that the difference in digestibility between AHBP-amylose complexes and MHBP-amylose complexes was attributed to the type and content of the main monomer phenol, which were consistent with Wang and Li et al. [42,43]. Moreover, adding 1% of ferulic acid and catechin could significantly delay starch digestion, i.e., we can achieve health benefits with low levels of ferulic acid and catechin. 

All in all, adding ferulic acid and catechin significantly reduced the RDS content and increased the RS content of amylose as expectedly. Considering the results of HBP-starch complexes, we attribute this to the complexation of ferulic acid, catechin with amylose, which might change the crystal structure of starch to form a more ordered structure, and then increase the resistance of starch to enzymatic hydrolysis [11]. Therefore, crystallinity and thermal properties of the complex between monomer phenol and amylose were further investigated to prove this hypothesis.

#### 3.6.2. Crystallinity and Thermal Properties of Amylose Compound with Main Monomer Phenol in Highland Barley

The effects of different types and additions of main monomer phenol on thermal properties of phenol-amylose complexes were shown in Figure 3D. ∆H represents the energy required to disrupt the ordered structure of starch during gelatinization [18]. Except for the 5% ferulic acid-amylose complex, the ∆H of other complexes was significantly lower than that of native amylose. It might be explained that the gelatinization and freeze-drying reduced the thermal stability of starch [44]. In addition, the ∆H of the same monomer phenol-amylose complexes increased gradually with the increase of the monomer phenols content from 1% to 5%, i.e., the interaction between ferulic acid, catechin and amylose were stronger with the rise of ferulic acid and catechin, this indicated the formation of a more orderly crystal structure and the requirement of higher gelatinization energy [45]. These results were in accordance with those of Zhang [30], who reported the ∆H of grape seed proanthocyanidin-potato starch complex increased significantly with the increase of grape seed proanthocyanidin binding amount.

**Figure 4:**
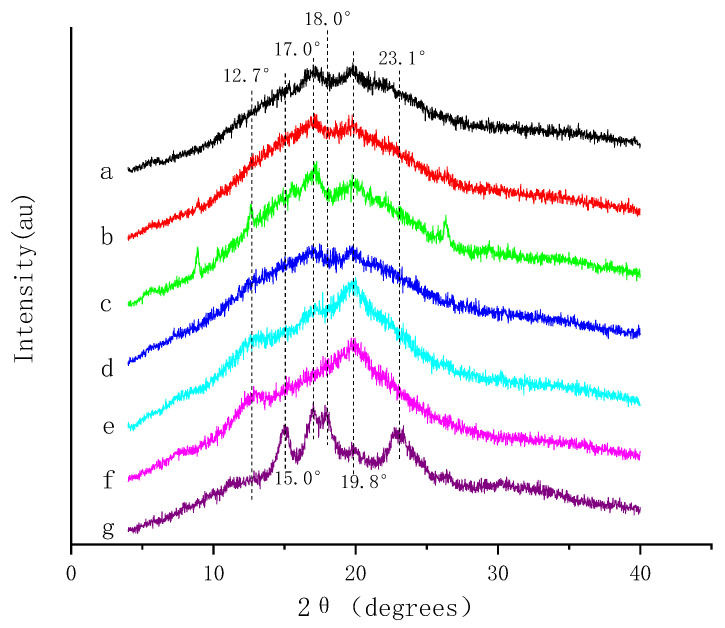
X-ray diffraction pattern of different standard substances and addition amounts polyphenol-amylose complexes. (a) 1% ferulic acid-amylose complex; (b) 3% ferulic acid-amylose complex; (c) 5% ferulic acid-amylose complex; (d) 1% catechin-amylose complex; (e) 3% catechin-amylose complex; (f) 5% catechin-amylose complex; (g) Amylose.

Consistent with the DSC results, XRD analysis indicated an interaction between ferulic acid-amylose and catechin-amylose (Figure 4). Amylose exhibited a typical A-type crystalline pattern with unresolved dual peaks at 17.0° and 18.0°, and two strong diffraction peaks at approximately 15.0° and 23.0°, respectively [36]. After compounding with ferulic acid and catechin, the unresolved dual peaks become weaker, and the peak at 23° and 15° disappear noticeably. The XRD diffraction pattern of all complexes showed pronounced peaks at 2θ of 19.8°, and some also had a diffraction peak at 12.7°, indicating the formation of a V-type complex [1,11]. The greater peak intensities at 19.8° among catechin-amylose complexes were observed with the increase in catechin incorporation rate, indicating the formation of a more ordered crystalline structure [46]. In addition, the ferulic acid-amylose complex also had a diffraction peak at 17.0°, whose intensity increased with increasing ferulic acid content. This indicated that a V-shaped complex partially formed between ferulic acid and amylose [47]. The interaction between starch and polyphenols would be affected by the molecular size, quantity and distribution of hydroxyl groups in phenolic compounds [8]. In our study, some amylose might not have large enough cavities and ferulic acid might not be hydrophobic enough for the spiral cavity of amylose, resulting in ferulic acid not enough to move into the cavity of amylose under hydrophobic action [13,27]. At this time, some ferulic acid might form a non-inclusion complex with amylose through hydrogen bonding [13].

## 4. Conclusions and Future Perspectives

This study investigated the structural properties and in vitro digestibility of HBP-starch complexes. HBP could form highly ordered V-type complexes with HBS and amylose through noncovalent bonds and then significantly reduce the content of RDS and increase the content of RS. In addition, the digestibility of polyphenol-amylose complexes varies with the type and content of monomer phenols. Both the ferulic acid-amylose and catechin-amylose complex effectively regulate the digestibility of amylose by altering the structure of amylose. It should be noted that other phenolic compounds interact with starch through noncovalent bonds to form complexes that change the digestive characteristics of starch.

The inhibitory mechanism of HBP on starch digestion was relatively complex. This study concluded that HBP could form V-shaped complexes with starch to inhibit starch digestion. It was still difficult to attribute this inhibition to a single mechanism, however. Whether the release of HBP in the complexes and other factors affect digestive enzymes during in vitro digestion remains to be further studied. Moreover, it is necessary to conduct an in vivo study of the dose–response relationship to quantitatively control postprandial blood glucose response. Given the worldwide prevalence of T2DM, our findings provide further support for the current recommendation that phenolic compounds including HBP can be used as functional components in starch-based food systems to delay starch digestion. 

## Figures and Tables

**Table 1:** Antioxidant activities, phenolic compound content, and main compositions in highland barley flour extracted by different solvents.

Index	Antioxidant Activities (μmol/g)		Phenolic Compounds Content (μmol/g)		Phenolic Compounds Composition (μg/g)		
	FRAP	TEAC	Total Phenolic	Total Flavone	Ferulic Acid	p-Coumaric Acid	Catechin
80% Methanol	14.62 ± 0.28 a	107.10 ± 0.29 b	4.99 ± 0.29 b	2.33 ± 0.35 b	0.848 ± 0.036 a	0.258 ± 0.021 a	0.942 ± 0.036 a
80% Ethanol	16.53 ± 0.26 a	106.73 ± 0.35 b	4.42 ± 0.27 b	3.23 ± 0.11 a			
80% Acetone	17.43 ± 1.44 a	116.30 ± 1.16 a	12.25 ± 1.13 a	3.38 ± 0.25 a	1.152 ± 0.010 b	0.214 ± 0.017 a	2.441 ± 0.069 b
Pure water	9.76 ± 1.32 b	57.17 ± 1.75 c	2.69 ± 0.19 c	0.99 ± 0.44 c			

FRAP: ferric reducing antioxidant power; TEAC: Trolox equivalent antioxidant capacity. Different letters in the same column represent significant differences (*p* < 0.05).

**Table 2:** Binding ability, 1047/1022 ratio, and digestive properties of highland barley polyphenol-starch complex extracted with different solvents.

Samples	Binding Ability(%)	1047/1022 (cm^−1^)	Digestive Properties		
			RDS (%)	SDS (%)	RS (%)
Amylose		1.378 ± 0.004 a	41.11 ± 0.99 a	50.18 ± 1.71 a	6.15 ± 0.81 ab
MHBP-amylose complexes	43.14 ± 1.81 a	1.536 ± 0.003 c	38.61 ± 0.09 ab	50.94 ± 1.63 a	7.64 ± 1.25 ab
AHBP-amylose complexes	56.36 ± 1.94 c	1.540 ± 0.002 c	36.17 ± 0.88 b	47.76 ± 3.32 a	13.27 ± 2.31 c
HBS		1.435 ± 0.005 b	39.99 ± 2.23 ab	52.54 ± 3.36 a	4.66 ± 2.21 a
MHBP-HBS complexes	44.74 ± 1.41 a	1.558 ± 0.003 d	38.16 ± 2.15 ab	49.18 ± 1.60 a	9.88 ± 0.93 bc
AHBP-HBS complexes	52.33 ± 0.90 b	1.558 ± 0.003 d	38.34 ± 1.61 ab	52.83 ± 2.75 a	6.24 ± 1.04 ab

MHBP: 80% methanol extracted highland barley polyphenols; AHBP: 80% acetone extracted highland barley polyphenols; HBS: highland barley starch; RDS: rapidly digestible starch; SDS: slowly digestible starch; RS: resistant starch. Different letters in the same column represent significantly different (*p* < 0.05).

## Data Availability

All data that support the findings of this study are available from the corresponding author on reasonable request.
